# Depression in Patients with Type II Diabetes: Case study at Diabetic Outpatient Clinic, in Samut Prakan

**DOI:** 10.5539/gjhs.v6n1p127

**Published:** 2013-10-27

**Authors:** Soontareeporn Thongsai, Suntaree Watanabenjasopa, Malinee Youjaiyen

**Affiliations:** 1 Department of Mental Health and Psychiatric Nursing, Boromarajonani College of Nursing, Bangkok, Thailand

**Keywords:** depressin diabetes patient, diabetes patient

## Abstract

This descriptive research studied the depression level of patients with diabetes type II at diabetic clinics in Samut Prakarn, and, identified the causes of severe depression in patients with type 2. There were 209 participants enrolled in the study. The samples were selected by opportunistic sampling technique. The data were collected from May 2013 to July 2013, using the CES-D questionnaire, with Cronbach’s coefficient alpha 0.82 and guidelines for interviews. Data were analyzed by descriptive statistics. Research Results: 1. 66 percent of participants had a depression score at a low level. 2. The CES-D showed that, 44 percent were unhappy and 38 percent did not feel that their life was enjoyable. 29 percent felt no hope for the future, 5.6 percent were easily upset and 8.3 percent suffered from insomnia and severe depression. 3. Half of the participants mentioned that troubled family relationships was a main cause of their depression, 42.9 percents felt worrying about their illness, 35.3 percent blamed over work and almost 15 percent identified loss of love as the cause of depression.

## 1. Introduction

Diabetes is a chronic, non communicable disease which is incurable. The complications of diabetes are a major public health problem in Thailand (Phong Ar-mon, 1999). It is estimated that some 194 million people worldwide, or 5.1% of the adult population, have diabetes and that this will rise to 333 million, or 6.3%, by 2025. (International Diabetes Federation [IDF], 2000 cited by Josling, 2001GBCHealth, 2013). Diabetes will become one of the biggest health problems in the 21st century, particularly in developing countries such as Thailand. A study conducted by the International Cooperation for the study of the epidemiology of diabetic patients (The International Collaborative study of Cardiovascular Disease in Asia) (Aekplakorn et al., 2003) found that diabetic patients in Thailand were *9.6 percent of the country's population or 2.4 million people*. In addition, according to health statistics of the year 2012 (Ministry of Public Health, 2012) the mortality rate of diabetes patient was 7,383 people per 100,000 population. Diabetes is the 4^th^ leading cause of death for non-communicable diseases. In addition, type 2 diabetes in the Tambon Health Promoting Hospital, Samut Prakarn ranked in second place for those receiving hospital care. In 2012, the number of diabetic patients who attended the outpatient clinic was 13,992 per year, with 904 admissions. It is self evident that diabetes is a major health problem and a growing problem. Thus diabetes will become a significant stressor on public health resources if the progression of the disease cannot be controlled, and a strain on society and the economy due to patients requiring continuous treatment throughout their life.

Diabetes is caused by the body’s loss of an ability to control the blood sugar level in the body (National Institute of Mental Health [NIMH], 2002), and the treatment is palliative care or the alleviation of the symptoms and the control of the spread of the disease (Um-porn Pan, Plern-ta, & Um-pon, 2001). Patients must modify their lifestyle in accordance with the disease, control diet, regularly medicate, and undertake intensive exercise and plan their self care (Vallarta, 1998) in order to prevent the complications.

Complications arising from diabetes, vascular complications of the eyes, kidneys and nervous system result in the disability and death. Nattikan (1999) found that patients diagnosed with type 2 diabetes, at 5 years, complications manifest and lead to a loss of enjoyment, suffering and premature death (Wan-dee and Jarintorn, 2541). Focused on the impact of diabetes on the body found that diabetic patients are unhappy, deteriorate, body systems fail leading to disability, and an inability to self care. Patients with diabetes have a worse health status and poorer quality of life than the others. Kinmond, McGee and Ashford (2003) found the illness affects the mental condition and emotional well being of patients through interrelated mechanisms.

Diabetes causes stress and leads to ennui, which adds to suffering from the illness. Diabetes is a threat to both and the physical and mental health of the patient. Concern about diabetes causes depression, from the stress of confronting the illness, treatment, and recovery (Griffiths, 2001; Sridhar & Madhu, 2002; Trigwell, 2001). Depression in patients with diabetes has found that 71 percent of patients were depressed. More commonly in patients with type 2 diabetes, and more frequently in patients over 40 years (Egede, Zheng, & Simpson, 2012), Diabetes is a disease that is associated with mental and physical behavior, emotional disorders are caused by physical disease and depression results due to stress that is associated with chronic illness. Depression can occur across all age group of people with diabetes. Patients are often treated for depression within six months after being diagnosed with diabetes (Delamater et al., 2001; Houiden, Jone, & Vallis, 2003).

This research investigates depression that occurs in insulin dependent diabetic patients. To contribute to the comprehensive care of patients: physically, mentally, emotionally and socially, to prevent of mental disorders and lead to a better quality of life for patients with type2 diabetes. It is consistent with the goal of treating diabetes to optimize care and the quality and the success of nursing practices to ensure the best outcome for patients

### 1.1 The Purpose of the Research

This research study was aimed to investigate patients with type 2 diabetes and the level of their depression and to study the causes of severe depression in patients with type 2 diabetes.

### 1.2 The Research Framework

This study used the conceptual framework of the Epidemiology Center for Epidemiologic studies-Depression scale (CES-D) (Radloff, 1997), which regards depression as a change of mood and behavioral expression in the form of negative emotions both towards oneself and the environment. Common characteristics include abnormal emotions and behaviors, for instance unhappiness, feel sad, irritability, feeling tired, frustrated, desperate for something to diminish or disappear, anorexic, fatigued, weak, suffer insomnia and dwell on feelings of worthless.

### 1.3 The Scope of the Research

This descriptive research recruited participants with type 2 diabetes attending the outpatient clinic in the Tambon Health Promoting Hospital, Muang district, Samut Pra-karn province from May to July 2013. Eligible participants selected by opportunistic sampling, 209 participants were finally enrolled in the study.

### 1.4 Participants: Inclusion Criteria

In accordance with the inclusion criteria of this research, participants those have been diagnosed with type 2 diabetes for more than 3 years (diagnosed by ICD 10), received the services from diabetic outpatient clinic at Tambon Health Promoting Hospital, Muang district, Samut Pra-karn province, adequate understanding of Thai and finally, sign the informed consent.

## 2. Research Methodology

### 2.1 2 Questionnaires Were Administered in this Study

1^st^ questionnaire- 2 parts

Part 1: There were 10 questions with multiple choices for demographic information such as, gender, age, marital status, education, religion, occupation, role in their family, complications, duration of illness, and the number of and frequency of admission to hospital.

Part 2: There were 2 open-end questions regarding the causes of depression such as “what is the most important that make participants feeling of depression?”

2^nd^ Questionnaire

Based on the level of depression in patients with diabetes, it was adapted from the Centre for Epidemiologic studies-Depression scale (CES-D) (Radloff, 1977) and translated into Thai language by Wilai and Phanom (1999, 2003). 20 questions, A 4 rating scale questionnaire, and 0 stands for rarely to 3 always, the point range is therefore 0 to 60. A score of 16 or more indicates depression. The Cronbach’s alpha reliability coefficient values were 0.82 and 0.85 respectively.

### 2.2 Ethical Considerations

At this stage, permission to recruit for this study from sought and given by the director of the Tambon Health Promoting Hospital in Samut Pra-karn such as, Tauy Ban Mai, Bang Muang and Taeparak, all staff involved were informed of the study. After obtaining ethics approval from the Boromarajajonani College of Nursing, Bangkok some modifications to improve clarity of the information sheet and consent form and feasibility of the proposed timetable were made in accordance with recommendations of the review committee. Individual consent was required for this study, and a letter of approval was presented to the directors of that Hospital, in which the study took place. Informed consent was obtained on the same day that the participants were recruited. Participants were informed that their participation in the study was voluntary. As the study began, patients were informed that they could refuse to answer any specific questions or they could terminate their participation at any point during the study period without giving reason.

## 3. Findings

### 3.1 Demographic Information

There were 209 participants enrolled in the study. The majority of the sample was female, 59.3% - 40.7% male. Ages ranged from 36 to 60 years old. 40.2% aged older than 60 years. 70.8% were married, 94.7% were Buddhists, 28.7% attained primary education, 39.2% were the head of the family (householders), 53.1% were not working, 62.2% had duration of illness for more than 5 years, 63.7% had complications and half of them had contracted hypertension, heart disease, stroke, and renal failure and foot ulcers, respectively (Table 1).

**Table 1:** Demographic characteristics of patients (N= 209)

Variables	Number	Percentage
Gender		
Male	85	40.7
Female	124	59.3
Aged		
36-60 yrs	125	59.8
More than 60 years	84	40.2
Religion		
Buddhist	198	94.7
Islam	6	2.9
Christian	5	2.4
Level of Education		
Primary school	60	28.7
Secondary school	54	25.8
Higher than secondary school	55	26.3
Bachelor degree	37	17.7
Postgraduate degree	2	1.0
Others	1	0.5
Marital status		
Single	18	8.6
Married	148	70.8
Separated	13	6.2
Divorced	8	3.8
Widow	26	12.4
Role’s in the family		
Householder	82	39.2
Member	127	60.8
Occupation		
Not working	111	53.1
Self employed	28	13.4
Employee	29	13.9
Farming	11	5.3
Civil servant	30	14.3
Duration of illness		
3-5 years	130	62.2
5 year up	79	37.8
Complications		
Yes	133	63.7
No	76	36.3

### 3.2 Patient’s Level of Depression

Most of the patients were mildly depressed at 63.2%, moderately depressed at 16.7% and severely depressed at 20.1 percent (Table 2).

**Table 2:** The number and percentage of the sample separated by level of depression (N= 209)

Level of depressed	Number	Percentage
Mild	132	63.2
Moderate	35	16.7
Severe	42	20.1

### 3.3 The Number of Patients with Type 2 Diabetes Having Positive and Negative Feelings

The study shows that feeling positive over a week that 44.4% reported feeling happy, 35% feeling happy sometimes, 40.6% thought that they were good in comparison to others. 42.8% had that feeling sometimes, 38.3% did not feel that their life is enjoyable and 38.3 percent felt enjoyment rarely and 28.9% felt hopelessness, 32.8 percent sometimes held hope for the future.

Regarding the negative feeling from the questionnaires, 10.0% always felt easily frustrated and 11.7% often felt easily frustrated. 16.1% often experienced difficulty sleeping, 8.3% always felt depressed and 17.2% suffered from a loss of appetite quite often.

### 3.4 Causes of Severe Depression in Patients with Type 2 Diabetes

34 people had severe depression with 4 main reasons given that cause depression.

3.4.1 Relationships within the family are not smooth.50 percent of participants indicated that family relationships are a cause of depression. Especially problem with their spouse, divorce may follow conflict and concern about the illness.

3.4.2 42.9% concerned about their illness leading to problems with depression. Most of the participants had complications such as, hypertension, heart disease, and some paralysis.

3.4.3 A heavy work load. 35.3% of participants identified that they had to many responsiblitiess and duties and this fact leads to depression and ennui,

3.4.4. The loss of the ones they loved. Participants stated that anxiety about the loss of the ones they loved was the cause of their depression at 14.7%.They feared facing the future alone.

## 4. Discussion

The survey approach provided descriptive information about the characteristics of this population. The objective was to study the number of patients with depression at different levels. And study the causes of depression in patients who underwent non- insulin dependent diabetes mellitus treatment at Tauy Ban Mai, Bang Muang and Taeparak, the Tambon Health Promoting Hospital in Samut Pra-karn.

### 4.1 Demographic Information

The study had 209 people in total, the majority were female (Table 1), consistent with studies on the incidence of diabetes. In general, the rate of diabetes in women is twice that in men (Department of Health, 2013 Sowattanangoon, 2009). The study by Wisutimakol (Wisutimakol, 1999) found that patients with diabetes, NIDDM were female and at approximately twice the incidence in males. For those in middle age, 36-60 years, the rate of type 2 diabetes is found to be highest in adults aged 40 years and over (Boon-Nak, 1999) and Brown (Brown, 2002) found that risk factors for people to develop type 2 diabetes includes being older than 45 years. Consistent with the study of the (Inlamphan, 2006) who found that those aged 40 years and above were at risk for type 2 diabetes and more likely to have diabetes. In terms of depression, a study by the International Institute of Mental Health of depression and diabetes (NIMH, 2002) found that 90% of patients with type 2 diabetes were aged 40 years or older (Boon-Nak, 2003)

In term of the duration of illness for participants with type 2 diabetes it was found that more than half of participants had been diagnosed with type 2 diabetes for at least 1-5 years which was in accordance to the previous study of Pong Ar-mond (1999). This supports the fact that diabetes type 2 is a chronic disease that requires treatment and attendance at a clinic for the rest of their life.

### 4.2 Number of Patients Separated by Level of Depression

The results showed that most of the participants have minor depression (Table 2), given that majority of them have had the illness for more than 3 year so, they may have learnt to adapt to their illness and accepted the reality and can lead normal lives, all participants were receiving the services from the clinics. In the other word, they were had a chance to discuss about their problems with others those had the same illness. Therefore, the feeling that they were not only person that ill with diabetes type 2, but on the other hand could make friends through the illness that they can consult, feel relaxed with, and accept and adjust themselves to get along with their illness.

This result accords with the study of Bill (1996) on the adaption of patients confronted with chronic illness. Adaption has 5 phases. Phase I patient refuses to accept the illness’s reality (denial). Phase II feeling anger (anger) due to the recognition that they were actually ill. Phase III. Depression, in this phase patients try to do everything to avoid of their sickness but cannot succeed. Phase IV negotiating or bargaining with the illness to improve the situation and Phase V a period of acceptance of the illness. The phases of adaptation of patients to chronic illness may take from 6 to 12 months. Therefore, the participants in this study have been through the five phases and are accepting of their illness.

Further the participants in this study having low level depression may be because depression is a mood condition that can happen to everyone being faced with disappointment, exposed to events or coping with the trauma of loss. Such depression is usually transitory, and may occur numerous times in life. Or in the other words is that participants soon accept their condition (Manasurakarn, 1998; Germain, 2002; Ehrlich, 2000). Results were consistent with the previous study of Soontareeporn (Thongsai, 2012) who stated that factors affecting to the health performance for patient with type2 diabetes were having gone through a period of adaptation once they have been sick for more than 5 years.

### 4.3 Number of Patients with Type 2 Diabetes Having Positive and Negative Feelings According to the Depression Questionnaire

From the positive perspective of the questionnaire, most participants were concerned that they were unhappy and did not think that their ability were as good as others (Table 3) due to their illness being incurable and having to maintain their treatment for all of their life. Additionally participants had complications associated with the illness and these cause stress as they feel that their illness is a burden to other people in the family and attributed the illness as the cause of the unrelated problems. Finally, an inability to solve the problem by themselves becomes a chronic stressor. It was in consistent with the concept in Lazarus and Folkman (1984) that people were stressed when factors producing the stress could not be control such as, those faced with illness, family problems which leads to psychological problems, stress, anxiety, fear and desperation. If a patient cannot adapt mental disorders and the emotional problems will impact on their mind. Associated feelings of desperation, frustrated by a lack of attention to the environment may cause to lack of the reasoning (Chaveewan, 1998).

**Table 3:** The number and percentage of patient’s feelings (N = 209)

Feeling	Number (percent)			
	Never	Sometimes	Quite often	Always
**Positive Feeling**				
1. I am happy	80(44.4)	63(35.0)	23(12.8)	14(7.8)
2. I am good comparable to others	73(40.6)	77(42.8)	15(8.3)	15(8.3)
3. I feel that my life is enjoyable	69(38.3)	69(38.3)	21(11.7)	21(11.7)
4. I have hope for the future	52(28.9)	59(32.8)	20(11.1)	49(27.2)
**Negative Feeling**				
5. I am easily frustrated	68(37.8)	73(40.6)	18(10.0)	21(11.7)
6. I feel bored of food	71(39.4)	68(37.8)	31(17.2)	10(5.6)
7. I have difficulty sleeping	76(42.2)	60(33.3)	29(16.1)	15(8.3)
8. I feel depressed	95(52.8)	56(31.1)	14(7.8)	15(8.3)
9. I find it hard to concentrate on things	111(61.7)	47(26.1)	18(10.0)	4(2.2)
10. I cannot solve problems with the support from family and friends	110(61.1)	44(24.4)	16(8.9)	10(5.6)
11. I am quiet	101(56.1)	40(22.2)	31(17.2)	8(4.4)
12. I am always crying	126(70.0)	38(21.1)	5(2.8)	11(6.1)
13. I feel hopeless in my life	126(70.0)	35(19.4)	6(3.3)	13(7.2)
14. I feel everything that I do works out wrong	127(70.6)	33(18.3)	15(8.3)	5(2.8)
15. I feel afraid	135(75.0)	31(17.2)	9(5.0)	5(2.8)
16. I feel that I am isolated	126(70.0)	30(16.7)	15(8.3)	9(5.0)
17. I am unhappy	133(73.9)	30(16.7)	9(5.0)	8(4.4)
18. I feel that some people around me do not like me	148(82.2)	26(14.4)	4(2.2)	2(1.1)
19. People are not friendly	145(80.6)	25(13.9)	7(3.9)	3(1.7)
20. I feel my life is going downhill	146(81.1)	17(9.4)	6(3.3)	11(6.1)

Focussing on the negative feelings the participants endorsed, 11.7% often felt frustrated (Table 3), which can arise with all illness, whatever the cause of the disease. The illness affects the physical and mental life of the patient as body and mind are interrelated as a mechanisms (Am-paipan, 2000), so that diabetic patients were feeling frustrated and stressed because of their disease together with a change in their life style to control the sugar level and these changes influence the mind. As, the study of Lustman and Anderson (2002) said that diabetes is a disease that depends on the patient behavior and mental status. So patients are stressed and irritated easily. The study found that 8.3 percent of participants had insomnia and depression often (Table 3), even the symptoms of diabetes can lead to insomnia and depression. The diagnostic criteria of DSM-IV stated that major depressive disorders, include sleep disorders, insomnia, and sleep a lot, or altered sleep pattern. All of these symptoms act and lead to depression. The effects sleep deprivation leads to fatigue and weakness, short term memory loss and learning difficulties, increased anxiety, and finally a diminished quality of life (Siriporn, 2003).

### 4.4 Causes of Depression

According to interview of those having severe depression, 34%, half of them stated that cause of their depression is disturbed relationships within their family. Divorce and problems with their spouse were the cause of conflicts within the family. It also found that the situation in the family especially the relationship between wife and husband the greatest cause of chronic stress, feeling discouraged, and a sense of hopelessness. On the other hand, if the relationship between husband and wife is supportive and there is family stability, a positive situation enables the family members to progress and achieve success in life. Whereas, if the relationship that occur in their family is not good they become stressors and obstruct the patient effort at behavioural modification with regard to their illness. So whether it is a man or woman both will need to have a strong understanding and supportive partner to cope together with the situation of chronic illness. Given that the illness is permanent, disabilities remain and the pathology the opportunity to be able to get back to normal is problematic. So they will need some support from the family (Wan, 2001).

42.9% of participants were worried about their illness. In addition, we found that they had complications such as, hypertension, heart disease and paralysis and these may be a burden to the family, feel loss of their function and importance, hopeless for the treatment. The illness soon affects other issues for instance, some people have more responsibility to take care of, an extra work, being the bread winner of the family, and stress from work. The unrelenting nature of the illness leads to difficulty in their life and an accumulation of stress and aggravation, leading to mental disorders and finally severe depression.

The study noted that various environmental factors become important once participants confront their difficult condition in life such as, a failure to find employment and other economic problem. These factors act as a stimulating factor for the onset of depression (Um-pornPan, 2000). It was in consistent with studies of Colun (2004) that depression and diabetes were found that apart from the strain of the disease or the treatment of disease there were the other factors that cause depression in patients with type 2 diabetes, including the effects of the treatment and decline of the social role.

### 4.5 Implications for Future Research Endeavours

The limitation of this study was conducted at a particular setting located in the rural area of Muang district Samut Prakarn, Thailand. This may affect the interpretability of the study in that, although the researcher was able to recruit a wide variety of participants, the outcome may not be extrapolated to a non rural setting. The findings from this trial are therefore limited to people living in such area that have similar characteristics to this study.

## 5. Researchers have suggested in the literature as follows

1. A comparative study of depression in patients with type 2 diabetes and other chronic diseases.
2. Study patient insights into the experience of depression, in patients with type 2 diabetes, to achieve a deeper understanding of the problem.
